# Synthesis, Properties and Biological Activity of Organotitanium Substituted Heteropolytungstates

**DOI:** 10.1155/MBD.2001.179

**Published:** 2001

**Authors:** Xiaohong Wang, Jianxin Li, Jianghua He, Jingfu Liu

**Affiliations:** Department of Chemistry Northeast Normal University Changchun 130024 China

## Abstract

Six new compounds [(CpTi)X, Cp=ϋ^∇^-c_∇_H_∇_, X=Ge, Ga, B) have been prepared and their Keggin structures determined by elementary analysis, IR, UV, ^∞^H NMR  and ^∞∀∊^WNMR spectrometry. The results show that the complexes retain Keggin structure. The complexes exhibit antitumoral activity *in vitro* as shown by MTT experiment.


### SYNTHESIS, PROPERTIES AND BIOLOGICAL ACTIVITY OF ORGANOTITANIUM SUBSTITUTED HETEROPOLYTUNGSTATES

Xiaohong Wang, Jianxin Li, Jianghua He and Jingfu Liu*

Department of Chemistry, Northeast Normal University, Changchun, China.P.R. 130024
<litterhongsd@163.net>

### ABSTRACT:

Six new compounds $[(CpTi)XW_{11}O_{39}]^{n-}$ ($Cp=\eta^5-C_5H_5$, X=Ge, Ga, B) have been prepared and their Keggin structures determined by elementary analysis, IR, UV, $^1H$ NMR and $^{183}W$ NMR spectrometry. The results show that the complexes retain Keggin structure. The complexes exhibit antitumoral activity in *vitro* as shown by MTT experiment.

### INTRODUCTION

Our recent work involves studies in both the antitumoral activity and catalytic activity of substituted heteropolyorganometallic complexes. Polyoxometalates (POMs) have attracted much attention because of their potential applications in catalysis, medicine and material science [1-3]. Hill has pointed out that the versatility of the POMs and grafting organic and organometallic groups on to the polyoxometalate surface can significantly increase their catalytic or medical applications. POMs containing a $[CpTi]^{3-}$ group show high anti-HIV activity [4] but, to the best of our acknowledge, there is no report on the antitumoral activity of organotitanium substituted heteropoly complexes. As part of our continuing investigation of POMs containing organometallic groups, we have systematically synthesized the $[CpTi]^{3-}$ substituted heteropolytungstates and investigated their antitumoral activity in *vitro.*

The title complexes $[(CpTi)XW_{11}O_{39}]^{n-}$ (X = Ge, Ga, B) are attractive targets for three reasons: first, $Cp_2TiCl_2$ is a more potent antiviral agent than other organotitanium compounds, but its insolubility in water makes it less useful in chemotherapy. So modifying $Cp_2TiCl_2$ into soluble complexes is a very attractive topic. Second, POMs containing titanium exhibit high antitumoral activity [5,6], so their investigation is very useful for determining the influence of the organometallic group on antitumoral or antiviral activity. Third, the synthesis of this kind of complexes can provide us with more compounds to study the medicinal applications of POMs.

### MATERIALS AND METHODS

2.1 Preparation of the compounds

$(C_5H_5)_2TiCl_2(Cp_2TiCl_2)$ was prepared according to ref. [7]

$K_8[GeW_{11}O_{39}].13H_2O$, $K_9[GaW_{11}O_{39}]\cdot xH_2O$ and $K_5Na_4[BW_{11}O_{39}]\cdot 13H_2O$ (abbreviated as $GeW_{11}$, $GaW_{11}$, $BW_{11}$) were prepared according to ref. [8] and identified by IR spectroscopy.

2.2 Preparation of $(Me_4N)_3H_2[(CpTi)GeW_{11}O_{39}]\cdot 11H_2O$

A solution containing 2.5 mmol (0.622 g) $Cp_2TiCl_2$, 3mmol acetylacetone and 15cm$^3$ $H_2O$ was stirred for about 4h to form a clear scarlet solution, to which was quickly added by 2.5mmol (7.5 g) of powdered $GeW_{11}$. After 30min of vigorous stirring the color of the solution turned from red to yellow, and the undissolved residue was filtered off. $Me_4NBr$ was added to the filtrate in small portions until no more precipitate was formed. The resulting yellow solid was recrystallized from hot water and dried under suction, giving a yield of 3.3g.The analytical data are summarized in Table 1.

2.3 *Preparation of* $(Me_4N)_aH_b[(CpTi)XW_{11}O_{39}]\cdot nH_2O$ *(X=Ga, B)*

$(Me_4N)_aH_b[(CpTi)XW_{11}O_{39}]\cdot nH_2O$ was prepared analogously. Powdered 2.5mmol $GaW_{11}$ or $BW_{11}$ was added to a solution of $Cp_2TiCl_2$ (2.5mmol). $Me_4NBr$ was used to precipitate out the product. Yield: 2.9g for $(CpTi)GaW_{11}$ and 3.6g for $(CpTi)BW_{11}$.

$(Bu_4N)_aH_b[(CpTi)XW_{11}O_{39}]\cdot nH_2O$ (X = Ge, Ga, B) were prepared similarly to the $Me_4N$ salts, except that powdered $Bu_4NBr$ was added to precipitate the $Bu_4N$ salts and recrystallized from hot $CH_3CN$.

2.4 *Antitumoral activity*

The antitumoral activity of POMs on two types of the human cancer cells was tested by the MTT experiment as described in ref. [9].

2.5 *Physical measurements*

$^1H$ NMR spectra were recorded on a Brucker AC-80 spectrometer. $^{183}W$ NMR were recorded on a Unity-

400 spectrometer. IR spectra were recorded on an Alpha Centauri FTIR spectrometer (4000-200cm$^{-1}$ range) as KBr pellets. UV spectra were recorded on a Beckman Du-8D spectrometer (solvent $H_2O$). Polarograms were obtained using a 384B polarographic analyser.

W, Ti, Si, Ge were determined by a Leeman Corporation inductively coupled plasma (ICP) emission spectrograph. C, N, H were determined using a PE-2400 analyzer. Water content was determined by thermogravimetry.

**Table 1 Analytical data for compounds (%)**

| Compounds | X | W | Ti | $H_2O$ | C | H | N |
|---|---|---|---|---|---|---|---|
| $(Me_4N)_3H_2[(CpTi)GeW_{11}O_{39}]\cdot 11H_2O$ | 2.32 | 63.11 | 1.51 | 5.97 | 6.20 | 1.39 | 1.33 |
| | (2.24)* | (62.16) | (1.47) | (6.08) | (6.27) | (1.32) | (1.29) |
| $(Me_4N)_4H_2[(CpTi)GaW_{11}O_{39}]\cdot 10H_2O$ | 2.00 | 60.32 | 1.50 | 5.31 | 7.67 | 1.61 | 1.71 |
| | (2.12) | (61.17) | (1.45) | (5.44) | (7.62) | (1.66) | (1.69) |
| $(Me_4N)_4H_2[(CpTi)BW_{11}O_{39}]\cdot 13H_2O$ | 0.39 | 60.87 | 1.51 | 7.19 | 7.58 | 1.64 | 1.72 |
| | (0.33) | (61.26) | (1.45) | (7.08) | (7.63) | (1.66) | (1.69) |
| $(Bu_4N)_3H_2[(CpTi)GeW_{11}O_{39}]\cdot 1H_2O$ | 2.00 | 56.13 | 1.30 | 0.40 | 7.79 | 3.29 | 1.14 |
| | (2.04) | (56.54) | (1.34) | (0.50) | (7.77) | (3.21) | (1.17) |
| $(Bu_4N)_5H[(CpTi)GaW_{11}O_{39}]$ | 1.77 | 51.24 | 1.11 | | 25.20 | 4.57 | 1.68 |
| | (1.73) | (50.07) | (1.19) | | (25.24) | (4.60) | (1.73) |
| $(Bu_4N)_4H_2[(CpTi)BW_{11}O_{39}]\cdot 1H_2O$ | 0.24 | 53.11 | 1.23 | 0.40 | 22.10 | 4.08 | 1.52 |
| | (0.29) | (53.83) | (1.28) | (0.48) | (22.02) | (4.01) | (1.49) |

*Calculated values

### RESULTS AND DISCUSSION

3.1 *The preparation*

In the previous report [10], the synthesis of $K_7Na_2[(CpTiOH_2)_3P_2W_{18}O_{68}]\cdot 15H_2O$ was carried out by reacting $CpTiCl_3$ with $Na_8HPW_9O_{34}\cdot xH_2O$ in water to obtain only low yield. So we changed the synthetic method as described in the preparation section. The synthesis of the $[(CpTi)XW_{11}O_{39}]^{n-}$ complexes may be represented by the following equations:

$Cp_2TiCl_2$+Hacac –> $[Cp_2Ti(acac)]^+$

$[Cp_2Ti(acac)]^+ + XW_{11}O_{39}^{(n+3)-}$ –> $[(CpTi)XW_{11}O_{39}]^{n-}$

3.2 *IR and U.V. spcetra*

The observed frequencies and tentative assignments of the main IR and UV bands of the title complexes are given in Table 2.

Compared with $XW_{12}O_{40}^{n-}$, the IR spectra of the substituted heteropolyanions still have the four characteristic peaks of $\nu_{as(W\text{-}Od)}$ (Od, terminal oxygen), $\nu_{as(W\text{-}Ob\text{-}W)}$, $\nu_{as(W\text{-}Oc\text{-}W)}$ (Ob, Oc, bridging oxygen) and $\nu_{as(X\text{-}Oa)}$ (Oa, central oxygen), indicating that the new complexes retain the basic frame of the Keggin structure. However, the terminal oxygen vibrations for $[(CpTi)XW_{11}O_{39}]^{n-}$ are shifted to low energy relative to the terminal oxygen vibration for $XW_{12}O_{40}^{n-}$. This is due to an increase of negative charge on the polyanion. The $\nu_{as(W\text{-}Oc\text{-}W)}$ of the complexes exhibit splitting, most probably resulting from the change of anion symmetry[11].

The IR spectra display a single, sharp absorption at 1469cm$^{-1}$ in the 1300-1500cm$^{-1}$ region. This feature is characteristic of the C-C stretch for a $\eta^5$-$C_5H_5$ ligand bonded to Ti [10]. At 3032, 3038cm$^{-1}$ in the 3030-3040cm$^{-1}$ region, the C-H stretches shows the existence of $\eta^5$-cyclopentadienyl ligand.

The title anions absorb at ca. 200±2 nm and 260±2 nm. The first band at higher energy has been assigned to the $O_{b/c}$–>W charge transfer band. The second band at lower energy is characteristic of 12-heteropolytungstates and has been attributed to the $O_d$–>W charge-transfer band.

**Table 2 The IR (cm$^{-1}$) and UV(nm) data of compounds**

| Anions | IR | | | | $\lambda_{max}$ | |
|---|---|---|---|---|---|---|
| Anions | $\nu_{as(W\text{-}Od)}$ | $\nu_{as(W\text{-}Ob\text{-}W)}$ | $\nu_{as(W\text{-}Oc\text{-}W)}$ | $\nu_{as(X\text{-}Oa)}$ | $O_d$–>W | $O_{b/c}$–>W |
| $(CpTi)GeW_{11}$ | 963 | 870 | 732,667 | 817 | 202 | 262 |
| $(CpTi)GaW_{11}$ | 942 | 871 | 739,661 | 563 | 198 | 260 |
| $(CpTi)BW_{11}$ | 945 | | 826,782 | 893 | 200 | 257 |
| $GeW_{12}O_{40}$[14] | 979 | 880 | 769 | 823 | | |
| $GaW_{12}O_{40}(H)$ | 970 | 890 | 780 | 560 | | |
| $BW_{12}O_{40}$ | 950 | | 817 | 900 | | |

### 3.3 *$^{1}H$ NMR and $^{183}W$ NMR spectra*

The $^{1}H$ NMR and $^{183}W$ NMR spectra are listed in Table 3.

**Table 3 The $^{1}H$ NMR and $^{183}W$ NMR data (ppm) of the compounds**

| Anions | $^{1}H$ | $^{183}W$ |
|---|---|---|
| $(CpTi)GeW_{11}$ | 6.52 | -59.61, -72.15, -76.99, -85.61, -95.61, -108.34 |
| $(CpTi)GaW_{11}$ | 6.53 | -59.09, -73.20, -96.90, -105.74, -124.96, -135.77 |
| $(CpTi)BW_{11}$ | 6.52 | -113.68, -115.53, -116.04, -130.76, -166.17, -174.74 |

The $^{1}H$ NMR spectra of the title complexes display a $\eta^{5}$-$C_5H_5$ resonance at *ca.* 6.52ppm.The $^{183}W$ NMR spectra of $CpTiXW_{11}$ exhibit six resonances.

In the well-known $T_d$ Keggin structure all the tungsten atoms are identical, as shown by a singlet in the $^{183}W$ NMR spectra. If one or more of the W-centered octahedral units are removed from a Keggin anion, lacunary anions are formed. For example, removal of a $[WO]^{4+}$ unit from $\alpha$–$[X^{n+}W_{12}O_{40}]^{(8-n)-}$ results in $\alpha$–$[X^{n+}W_{11}O_{39}]^{(12-n)-}$ which features one structurally unique W atom and five inequivalent pairs of W atom as imposed by Cs point symmetry, and the expected six lines are obtained in the $^{183}W$ NMR spectra .The $^{183}W$ NMR spectra of $(CpTi)XW_{11}$ confirm that the substituted $[CpTi]^{3+}$ group occupies an empty octahedral site of a $XW_{11}$ anion and forms a polyanion with a Keggin structure.

### 3.4 *Electrochemistry*

The redox proprieties of $(CpTi)XW_{11}$ were investigated by polarography. The half-wave potentials in different pH values are listed in Table 4. It can be seen that: (1) The reduction process of $(CpTi)XW_{11}$ anions involve the reduction of titanium and tungsten. The first peak is assigned to the conversion of Ti (IV) to Ti (III) [12]. (2) The latter two polarographic peaks are assigned to the conversion of W (VI) to W (V), showing that the title anions undergo a two-step tungsten reduction process. They are pH-dependent reduction waves. And straight lines are obtained by logarithmic analysis of the polarographic wave. The number of electrons involve in the reduction can be calculated from the slope of the straight lines, showing that $(CpTi)XW_{11}$ has undergone a two-step, two-electron tungsten reduction. (3)$E_{1/2}$ shifts to negative potentials with increasing pH of the solution, implying that the reduction of polyanions is accompanied by protonation. From the slope of $E_{1/2}$ *versus* pH, it can be calculated that when $n = 2$, $m = 2$ (n and m stand for the electron and proton number, respectively) for the reduction of tungsten, two protons must be added to gain two electrons while the polyanion is reduced; this prevents charge build-up on the anion, which would otherwise be destabilized.

**Table 4 Polarographic half-wave potential (v) for the title compounds***

| Anions | $pH_{4.7}$ | $pH_{5.2}$ | $pH_{5.7}$ |
|---|---|---|---|
| $(CpTi)GeW_{11}$ | -0.472 | -0.474 | -0.475 |
| | -0.740 | -0.783 | -0.819 |
| | -1.020 | -1.056 | -1.099 |
| $(CpTi)GaW_{11}$ | -0.463 | -0.471 | -0.482 |
| | -0.654 | -0.702 | -0.744 |
| | -0.841 | -0.879 | -0.901 |
| $(CpTi)BW_{11}$ | -0.474 | -0.480 | -0.487 |
| | -0.843 | -0.884 | -0.922 |
| | -0.986 | -1.025 | -10.69 |

* All polarography in $1.0 mol\cdot dm^{-3}$ acetate buffer solutions with $5x10^{-4} mol\cdot dm^{-3}$ $Me_4N$ salts of complexes

### 3.5 *Antitumoral activity*

The data summarized in Table 5 show that the title complexes display inhibitory action to two types of human cancer cells Hela and SSMC-7721 tumor cells.

### 3.6 *The stability of the POMs at the physiological condition*

In order to assess the stability of the complexes, they were treated in the same MTT experimental conditions but without adding cancer cells. After evaporation to dryness, IR and $^{1}H$ NMR spectra showed that the complexes retained the Keggin structure and the $[CpTi]^{3+}$ group was not eliminated from the $XW_{11}$ moiety demonstrating that they are stable under the evaluation conditions.

**Table 5 Inhibitory effect of the title complexes on tumor cells in *vitro***

| Compounds | Tumor cell | Dose($\mu g \cdot L^{-1}$) | Inhibitory rate | $IC_{50}$*($\mu g \cdot L^{-1}$) |
|---|---|---|---|---|
| $(CpTi)GeW_{11}$ | SSMC | 100 | 100 | |
| | | 10 | 15.4 | 47.8 |
| | | 1 | 1.1 | |
| | Hela | 100 | 93.2 | |
| | | 10 | 10.4 | 54.5 |
| | | 1 | 0 | |
| $(CpTi)GaW_{11}$ | SSMC | 100 | 100 | |
| | | 10 | 12.4 | 42.6 |
| | | 1 | 0.9 | |
| | Hela | 100 | 90.0 | |
| | | 10 | 9.4 | 48.3 |
| | | 1 | 0 | |
| $(CpTi)BW_{11}$ | SSMC | 100 | 100 | |
| | | 10 | 21.4 | 38.9 |
| | | 1 | 1.7 | |
| | Hela | 100 | 100 | |
| | | 10 | 16.9 | 43.6 |
| | | 1 | 0.8 | |

*The 50% inhibitory concentration ($IC_{50}$) is defined as the concentration that suppresses tumor cells by 50%.

### REFERENCES

[1] M. T. Pope, *Heteropoly and Isopoly Oxometalates*; Springer-Verlag, New York, 1983

[2] M. T. Pope and A. Muller, *Polyoxometalates: From Platonic Solids to Antiretroviral Activity*; Kluwer Academic Press, Dordrecht, The Netherlands, 1994

[3] M. T. Pope and A. Muller, *Angew. Chem. Int. Ed. Engl.*, **30**, 34 (1991)

[4] N. Yamamoto, D. Schols, E. De Clercq, Z. Debyser, R. Pauwels, J.Balzarini, H.Nakashima,M.Baba, M.Hoaoya, R. Snoeck, J.Neyts, G. Andtei, B.A.Murrer, B.Theobald, G.Bossard, G.Henson, M.Abrams, D.Picker, *Mol. Pharmacol.*, **42**, 1109 (1992)

[5] Y. Inouye, Y. Tokutake, Y.Seto, H.Hujita, A.Yamamoto, S. Nishiya, T. Yamase, S.Nakamura, *Antiviral Res.*, **20**, 317 (1993)

[6] D. L. Barnard, C. L. Hill, T. Gage, J.E.Matheson , J.H.Huffamn,R.W.Sidwell, M.I.Otto, R.F.Schinazi *Antiviral Res.*, **34**, 27 (1997)

[7] G. Wilkinson, J. M. Birmingham, *J. Am. Chem. Soc.*, **76**, 4281 (1954)

[8] H. Nobuynki, O. Yoshihiro, I. Toshiyuki, M. Yoshihisa, *Inorg. Chem.*, **33**, 1015 (1994)

[9] X. H. Wang, J. F. Liu, Y. G. Chen, Q. Liu, J. T. Liu, M. T. Pope, *J. Chem. Soc. Dalton Trans.*, 1139 (2000)

[10] W. H. Knoth, P. J. Domaille, R. D. Farlee, *Organometallics*, **4**, 62 (1985)

[11] C. Rocchiccioli-Deltcheff, R. Thouvenot,*J. Chem. Res., Synopses*, **2**, 46 (1977)

[12] J. F.Liu, L.Men, W.Y.Zhao, J.C.Li, B.L.Zhao, *Acta Chim. Sinica*, **53**, 46 (1995)

**Received: June 5, 2001 – Accepted: June 16, 2001 – Accepted in publishable format: October 16, 2001**

